# Prognostic Value of Procalcitonin, C-Reactive Protein, and Lactate Levels in Emergency Evaluation of Cancer Patients with Suspected Infection

**DOI:** 10.3390/cancers13164087

**Published:** 2021-08-13

**Authors:** Patrick Chaftari, Aiham Qdaisat, Anne-Marie Chaftari, Julian Maamari, Ziyi Li, Florea Lupu, Issam Raad, Ray Hachem, George Calin, Sai-Ching Jim Yeung

**Affiliations:** 1 Department of Emergency Medicine, The University of Texas MD Anderson Cancer Center, Houston, TX 77030, USA; pchaftari@mdanderson.org (P.C.); aqdaisat@mdanderson.org (A.Q.); 2 Department of Infectious Diseases, Infection Control and Employee Health, The University of Texas MD Anderson Cancer Center, Houston, TX 77030, USA; achaftari@mdanderson.org (A.-M.C.); iraad@mdanderson.org (I.R.); rhachem@mdanderson.org (R.H.); 3 School of Medicine, Lebanese American University, P.O. Box 36, Byblos, Lebanon; julian.maamari@lau.edu; 4 Department of Biostatistics, The University of Texas MD Anderson Cancer Center, Houston, TX 77030, USA; zli16@mdanderson.org; 5 Cardiovascular Biology Research Program, Oklahoma Medical Research Foundation, Oklahoma City, OK 73104, USA; Florea-Lupu@omrf.org; 6 Department of Translational Molecular Pathology, The University of Texas MD Anderson Cancer Center, Houston, TX 77030, USA; gcalin@mdanderson.org

**Keywords:** emergency department, infectious oncologic emergencies, procalcitonin, C-reactive protein, lactic acid, sepsis

## Abstract

**Simple Summary:**

Cancer patients are at increased risk of infections and related complications, including sepsis. We developed a scoring system for mortality prediction based on readily available clinical and laboratory data, including the quick sequential organ failure assessment (qSOFA) score, cancer subtype, and several laboratory markers (procalcitonin, C-reactive protein, lactate dehydrogenase, and albumin) that can be used in emergency departments for cancer patients with suspected infection. The prediction score, which stratifies patients into four different risk groups (from low risk to very high risk), achieved excellent performance in predicting 14-day mortality, with an area under the receiver operating characteristic curve value of 0.88 (95% confidence interval 0.85–0.91). The score was also effective in predicting intensive care unit admission and 30-day mortality.

**Abstract:**

Cancer patients have increased risk of infections, and often present to emergency departments with infection-related problems where physicians must make decisions based on a snapshot of the patient’s condition. Although C-reactive protein, procalcitonin, and lactate are popular biomarkers of sepsis, their use in guiding emergency care of cancer patients with infections is unclear. Using these biomarkers, we created a prediction model for short-term mortality in cancer patients with suspected infection. We retrospectively analyzed all consecutive patients who visited the emergency department of MD Anderson Cancer Center between 1 April 2018 and 30 April 2019. A clinical decision model was developed using multiple logistic regression for various clinical and laboratory biomarkers; coefficients were used to generate a prediction score stratifying patients into four groups according to their 14-day mortality risk. The prediction score had an area under the receiver operating characteristic curve value of 0.88 (95% confidence interval 0.85–0.91) in predicting 14-day mortality. The prediction score also accurately predicted intensive care unit admission and 30-day mortality. Our simple new scoring system for mortality prediction, based on readily available clinical and laboratory data, including procalcitonin, C-reactive protein, and lactate, can be used in emergency departments for cancer patients with suspected infection.

## 1. Introduction

The incidence of infection and sepsis is remarkably higher in cancer patients when compared with the general population [1,2,3]. Multiple factors, including cancer-related factors (e.g., leukemia-associated neutropenia), treatment-related factors (e.g., chemotherapy-induced neutropenia), and patient-related factors, account for the increased risk [3,4]. Roughly 60% of deaths in patients with hematologic malignancies and 50% in patients with solid tumors are a direct result of infectious complications [5,6,7]. Infections in cancer patients often occur acutely and may progress rapidly. Therefore, the emergency department (ED) is often the first clinical setting to which cancer patients present for diagnosis and management of infection/sepsis.

Biomarkers, including lactate, C-reactive protein (CRP), and others, play an important role in diagnosing sepsis and guiding its management [8,9,10]. Lactate is a metabolite generated from glycolysis, and accumulation in the blood reflects poor tissue perfusion and substantial hemodynamic compromise. In sepsis, lactate has been used as a prognostic [11,12,13], diagnostic [8], monitoring [13], and stratification biomarker [8,11,12,13]. CRP is an acute phase reactant, and increased circulating CRP levels follow rising levels of cytokines (e.g., interleukin-6, tumor necrosis factor-a), which are sustained in the course of sepsis. Plasma CRP > 62.8 mg/L is an optimal cutoff value for predicting high risk of death from sepsis [14]. Other biomarkers include procalcitonin, cluster of differentiation 64, and proadrenomedullin [15].

In recent years, procalcitonin has become a very important biomarker for bacterial infection and sepsis. Procalcitonin is a 116-amino acid protein with an approximate molecular weight of 14.5 kDa, and it is encoded by the CALC-1 gene on chromosome 11 [16,17]. It is a prohormone primarily produced by the C cells of the thyroid gland [17,18], and it has very low circulating concentrations (<0.1 ng/mL) in healthy humans [18]. In the event of a bacterial infection, procalcitonin production and release into the circulation is part of the systemic response to circulating cytokines and endotoxins [19]. The plasma concentration of procalcitonin correlates with the severity of infection and sepsis [19]. Procalcitonin levels are higher in systemic bacterial infection than in localized viral or bacterial infection [19], but procalcitonin levels may also be low early in Gram-positive bacterial systemic infection regardless of severity [20]. The top organism isolated from the blood culture of patients with low procalcitonin is *Staphylococcus aureus* [21]. False negatives, such as those associated with Gram-positive bacterial infections [20,21], as well as false positives [22,23,24], decrease the accuracy of plasma procalcitonin levels in predicting bacterial systemic infection. However, procalcitonin is more helpful in identifying Gram-negative bacterial systemic infections [25], and the accuracy of procalcitonin in predicting Gram-negative bacteremia in leukemia patients is quite good (area under the receiver operating characteristic curve (AUROC) = 0.779) [26]. Longitudinal assessment using serial procalcitonin measurements can predict prognosis and treatment efficacy in infections [27], and this assessment is used in antibiotic stewardship [28,29,30]. Procalcitonin is also used as an independent predictor of clinical deterioration in ED patients with suspected sepsis [31].

Early diagnosis and treatment significantly reduce sepsis mortality. When integrated with clinical information (e.g., clinical scores, such as the quick sequential organ failure assessment (qSOFA) score), biomarkers have great potential to improve diagnostic and prognostic assessment of patients [32,33]. Combining the qSOFA score with procalcitonin was shown to predict 28-day mortality among high-risk sepsis patients better than qSOFA alone [33,34]. Currently, our institution, which serves cancer patients specifically, has implemented an early sepsis alert warning system based on vital signs that suggest the presence of systemic inflammatory response syndrome. However, in the ED, infection in cancer patients may be in different stages and severity levels, and the assessment is cross-sectional. It is challenging to integrate the clinical and laboratory information available to the ED physician to guide clinical decisions for this patient population. The aim of the current study was to determine whether plasma levels of procalcitonin, CRP, and lactate, which are routinely measured in patients with suspected infection or sepsis in our ED, could be combined with clinical information to accurately predict mortality rates and risk-stratify cancer patients in the ED with suspected infection.

## 2. Materials and Methods

### 2.1. Study Participants and Data Collection

To assess the role of procalcitonin, CRP, lactate, and other clinical factors in the management of suspected infection in cancer patients, we conducted a retrospective cohort study. Our study was approved by the Institutional Review Board of The University of Texas MD Anderson Cancer Center, protocol number DR08-0066. This study included all consecutive patients with a known cancer diagnosis who presented to the ED between 1 April 2018 and 30 April 2019, and had procalcitonin levels measured during their ED visit. Patients younger than 18 years and those without a confirmed cancer diagnosis prior to the ED visit were excluded from the analysis (Figure S1).

### 2.2. Statistical Analysis

Descriptive statistics were used to summarize the main characteristics of the final cohort. Variations in lactate, CRP, and procalcitonin levels among patients with different qSOFA scores or different bacterial blood culture results (growth or no growth) were investigated. The variation of procalcitonin levels among patients with different cancer stages was also examined. The Wilcoxon-Mann-Whitney test or one-way analysis of variance followed by a post hoc Tukey test was used where appropriate to identify significant differences in inter-group comparisons. A two-tailed *p* value < 0.05 was considered statistically significant. All statistical analyses were performed using R software (version 3.5.1, The R Foundation, http://www.r-project.org, accessed date: 25 September 2018). Relevant packages used include randomForest, pROC, survival, survminer, ggplot2, and ggthemes.

Cancer patients are frequent visitors of the ED, and each visit is unique in terms of related clinical presentation and laboratory results. Therefore, we used the ED visit as the unit of observation. Death within 14 days was chosen as the primary outcome for the development of a prediction model. qSOFA scores were calculated for each visit by adding 1 point for each qSOFA parameter (altered mental status, systolic blood pressure ≤ 100, and respiratory rate ≥ 22), stratifying patients into one of two groups: not high risk for in-hospital mortality (qSOFA score: 0–1) and high risk for in-hospital mortality (qSOFA score: 2–3) [35,36,37]. Fourteen-day mortality rates were first examined for different qSOFA scores, after which a primary prediction model was built using a multivariable logistic regression model consisting of the three main studied biomarkers (procalcitonin, CRP, and lactate) combined with the qSOFA score. Variables included in the primary prediction model development phase were the clinical variables that have been previously shown to be associated with mortality in patients with infection/sepsis [8,9,38]. The AUROC metric was used to evaluate the predictive performance of classifiers of the primary model [39]. We then examined the possibility of improving the prediction model by adding cancer type, along with other clinical and laboratory factors that are readily collected in the ED. Investigated variables with more than 20% missing values were not included in further analysis, and missing values for the other predictor variables (≤20% missing values) were imputed using proximity from the R package “randomForest” (rfImpute function).

Cancer types were stratified according to risk of death within 14 days using a multivariable logistic regression model controlling for age and creatinine levels, and the model was further simplified by grouping cancer types with similar adjusted odds ratios (AORs) into one of three groups: high risk (AOR ≥ 2), intermediate risk (AOR between 1 and 2), and low risk (AOR ≤ 1). Random forest prediction of 14-day mortality was performed to identify clinical and laboratory variables with high relative importance as predictors of the outcome. The cancer type risk groups (high, intermediate, and low) and the variables with the top mean decrease in Gini from the random forest model were added to the primary prediction model, building the final multivariable logistic regression model. The Hosmer–Lemeshow goodness-of-fit statistic was used to calibrate the model. Repeated K-fold cross-validation was used to internally validate the model. AUROC was used to assess the prediction performance of the final prediction model, and the DeLong test was used to identify statistically significant differences between the models.

### 2.3. Construction and Evaluation of the Final Predictive Scoring System

Once the final prediction model was determined, laboratory variables were categorized using the current clinical cutoff point used in cancer patients or a new optimal cutoff point selected using the Youden index. Then, to construct the final prediction score, we assigned points to each predictor based on its beta coefficient (i.e., the beta coefficient for each predictor in the final prediction model was divided by the coefficient of the predictor with the smallest coefficient, rounding the result to the nearest integer). Patients were categorized into four risk groups using three different cutoff points of the prediction score. The cutoff points were chosen on the basis of pre-set criteria that the 14-day mortality rate was low (<1%), intermediate (1–10%), high (10–25%), or very high (>25%). We also evaluated the performance of the prediction score in predicting 30-day mortality and intensive care unit (ICU) admission, reporting the respective rates for each of the four risk groups. Figure S2 demonstrates the steps involved in the development of the final prediction score. Kaplan–Meier survival analysis followed by the log-rank test was used to estimate the difference in 3-month survival rates between the final prediction score risk groups. Patients who did not die within 3 months after presentation were censored at the time of their last known contact date, as were those who were lost to follow-up at 3 months (90 days) after the index ED visit. Univariate and multivariable Cox proportional hazards analyses were used to investigate the association between each risk group and survival duration, calculating the hazard ratio (HR) with a 95% confidence interval (CI) and controlling for age, sex, and race in the multivariable analysis.

## 3. Results

### 3.1. Patient Characteristics

The total number of unique cancer patients eligible for inclusion in our study was 3623 patients, who made 5118 unique ED visits (Figure S1). Seventy-two percent of the patients had only one ED visit during the period studied. The median number of days to ED revisit for the patients who had more than one visit was 36 days (interquartile range (IQR): 17–76 days). The demographic and clinical characteristics of the patients, as well as by ED visit, are shown in Table 1. The median age of the study population was 62 years, and 48.7% were female and 51.3% were male. Leukemia (16.6%), lymphoma (10.9%), breast cancer (9.1%), lung cancer (8.6%), and sarcoma (5.8%) were the most frequent cancer types. The 14-day mortality rates by qSOFA score for ED visits are shown in Table S1. Bacteremia was confirmed in 488 of the visits (9.5%). For most visits (4912 (96.0%)), the patient had a qSOFA score of ≤1, and of the 224 patients who died within 14 days of their ED visit, 187 (83.5%) had a qSOFA score of ≤1. A total of 4890 (95.5%) blood samples were collected and cultured; of these, 488 (10.0%) were positive. Of the positive cases, 254 were Gram negative and 234 were Gram positive.

### 3.2. Variation of Infection Biomarkers

**Figure 1:**
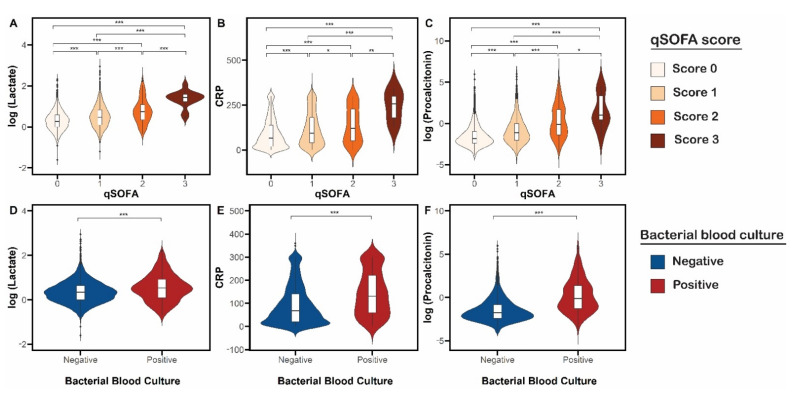
Variation of infection/sepsis biomarker levels stratified by quick sequential organ failure assessment (qSOFA) scores and bacterial blood culture results in cancer patients presenting to the emergency department with suspected infection. Upper panel: Distribution of (**A**) lactate, (**B**) C-reactive protein (CRP), and (**C**) procalcitonin levels by qSOFA score. A one-way analysis of variance followed by the post hoc Tukey test was used for statistical analysis. Lower panel: Distribution of (**D**) lactate, (**E**) CRP, and (**F**) procalcitonin levels for positive and negative bacterial blood culture results. The Wilcoxon-Mann-Whitney test was used for statistical analysis. * *p* < 0.05; ** *p* < 0.01; *** *p* < 0.001.

We examined the variation of lactate, CRP, and procalcitonin levels among patients with different qSOFA scores (Figure 1 and Table S2). All three biomarkers were significantly higher with each incremental increase in qSOFA score. For example, in patients with a qSOFA score of 3 compared with a score of 0, median lactate was 4.20 mmol/L compared with 1.30 mmol/L, median CRP was 257.60 mg/L compared with 67.10 mg/L, and median procalcitonin was 2.70 ng/mL compared with 0.16 ng/mL (*p* < 0.001 for all comparisons). Similarly, the three biomarkers were significantly higher among patients with positive blood culture (Figure 1 and Table S3). Median procalcitonin levels did not significantly differ among those with advanced/metastatic cancer (0.21 ng/mL, IQR: 0.1–0.62 ng/mL), localized/undetermined cancer (0.14 ng/mL, IQR: 0.07–0.35 ng/mL), and hematologic malignancy (0.21 ng/mL, IQR: 0.11–0.54 ng/mL; Figure S3). Median procalcitonin and lactate levels were significantly (*p* < 0.001) higher in patients with Gram-negative blood cultures (median procalcitonin [IQR] = 2.18 [0.48–6.28] ng/mL and median lactate [IQR] = 1.8 [1.3–2.8] mmol/L) when compared to patients with gram-positive blood culture (median procalcitonin [IQR] = 0.41 [0.20–1.60] ng/mL and median lactate [IQR] = 1.5 [1.1–2.2] mmol/L; Figure S4).

### 3.3. Logistic Regression Model Analyses

**Figure 2:**
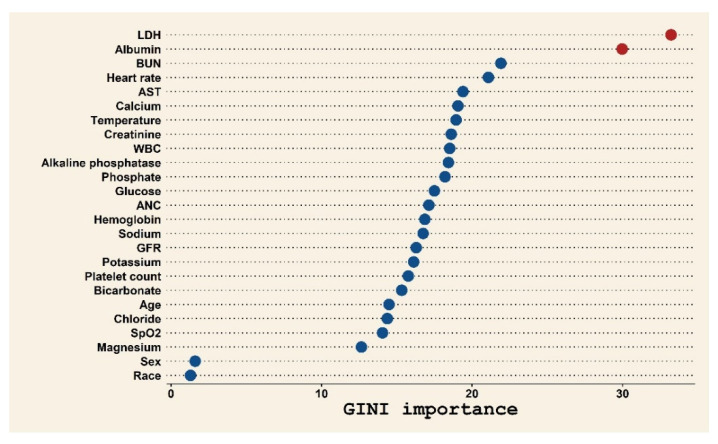
Classification of 14-day mortality using common clinical and laboratory variables among cancer patients presenting to the emergency department with suspected infection. Abbreviations: LDH, lactate dehydrogenase; BUN, blood urea nitrogen; AST, aspartate aminotransferase; WBC, white blood cell count; ANC, absolute neutrophil count; GFR, glomerular filtration rate; SpO2, oxygen saturation.

Multivariable logistic regression was used to build the primary prediction model, which had four components (qSOFA, procalcitonin, CRP, and lactate). This primary model yielded an AUROC of 0.83 (95% confidence interval 0.79–0.87) for predicting 14-day mortality (Figure S5). Stratification of cancer type according to risk resulted in three groups defined by AOR (Table S4): high-risk cancer (gastric, esophageal, hepatobiliary, and pancreatic cancer), intermediate-risk cancer (lung cancer, melanoma, colorectal cancer, urinary cancer, breast cancer, and gynecologic cancer), and low-risk cancer (all other types). For the other clinical and laboratory data predictor variables, random forest analysis identified two important laboratory components (lactate dehydrogenase and albumin) that can predict 14-day mortality (Figure 2). Therefore, cancer type risk group, lactate dehydrogenase, and albumin were added to the primary prediction model. The predictive performance of the resulting final prediction model improved (Figure S5), yielding an AUROC of 0.88 (95% confidence interval 0.85–0.91), which was significantly higher than in the primary prediction model (DeLong test, *p* = 0.044). Hosmer–Lemeshow *p* = 0.837 for the final prediction model, indicating no evidence of poor fit. Internal validation using K-fold cross-validation of the final prediction model showed a mean accuracy of 0.969 with a prediction error of 0.0274, with K = 10 (Figure S6).

### 3.4. Constructing and Evaluating the Scoring System

To build the prediction score system, the continuous variables were first categorized as stated in the methods section: procalcitonin ≥ 0.15 ng/mL, lactate ≥ 2.0 mmol/L, CRP ≥ 115 mg/L, lactate dehydrogenase ≥ 285 U/L, and albumin < 3.5 g/dL. A multivariable logistic regression final model was built using qSOFA score, cancer type risk group, and the established dichotomous laboratory variables. Based on the beta coefficients of the variables in this final model, a prediction score was built by assigning points to each variable by dividing the beta coefficient of the predictor by the beta coefficient of the predictor with the smallest coefficient (i.e., 0.367 for the intermediate cancer risk group), rounding the result to the nearest integer (Table 2). Calculating the score for each patient yielded good discriminatory partitioning of the 14-day mortality rates among groups (Table S5).

**Figure 3:**
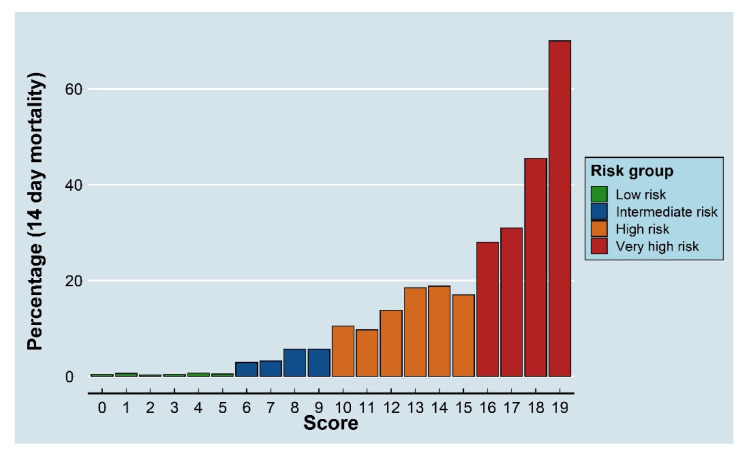
Fourteen-day mortality rates for different prediction scores and risk groups among cancer patients presenting to the emergency department with suspected infection.

**Figure 4:**
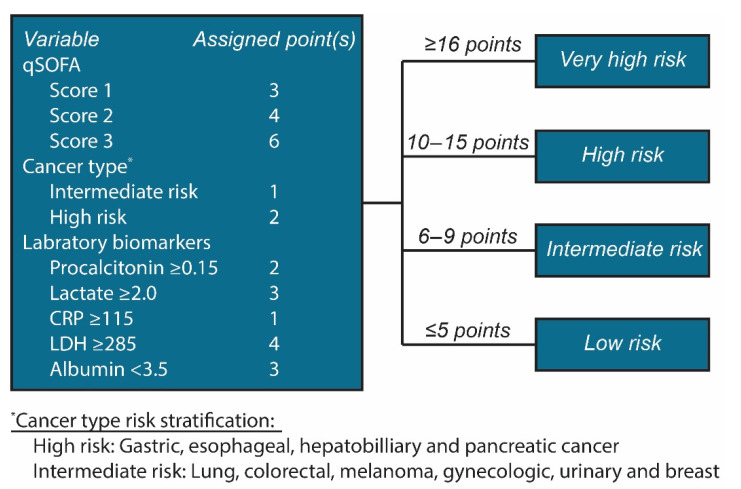
Algorithm of the final prediction score for 14-day mortality among cancer patients presenting to the emergency department with suspected infection. qSOFA, quick sequential organ failure assessment; CRP, C-reactive protein; LDH, lactate dehydrogenase.

Based on the pre-set criteria (see the methods section) and these results, the stratified risk groups and cutoff points were as follows: low risk: ≤5 points; intermediate risk: 6–9 points; high risk: 10–15 points; and very high risk: ≥16 points (Figure 3). Using these cutoff points, the 14-day mortality rates for each group were as follows: low risk 0.4%, intermediate risk 4.2%, high risk 14.2%, and very high risk 41.2% (Table S6). The final scoring system algorithm is shown in Figure 4.

Kaplan–Meier analysis showed a significant difference (*p* < 0.001) in the 3-month survival rate between the final risk groups (Figure S7). The poorest survival was observed in patients with high (HR = 8.81, 95% CI = 7.42–10.48, *p* < 0.001) and very high risk (HR = 24.90, 95% CI = 17.38–35.67, *p* < 0.001) in both the univariate and multivariable Cox proportional hazards analyses (Table S8).

We further tested the scoring system on two other outcomes: 30-day mortality and ICU admission. The score showed good performance in predicting both outcomes: patients in the low-risk group had a 1.7% 30-day mortality rate and a 0.4% ICU admission rate, whereas patients in the very-high-risk group had a 56.9% 30-day mortality rate and a 41.2% ICU admission rate (Table S7).

## 4. Discussion

Using data from cancer patients who presented to the ED of a comprehensive cancer center with suspicion of infection/sepsis, we devised a clinical prediction scoring system to stratify the risk of 14-day mortality among these patients (Figure 4). The major strength of our prediction model is that it combines clinical and laboratory factors with the patient’s cancer type to achieve a prediction score that is more suitable for cancer patients needing emergency care than the known scoring systems used in the general population. The prediction score achieved excellent performance in predicting 14-day mortality, with a rate of 0.4% for the low-risk group compared with 41.2% for the very high-risk group. The prediction score also showed excellent performance when tested for two other outcomes: ICU admission and 30-day mortality.

Multiple biomarkers have been used as diagnostic and/or prognostic biomarkers predicting outcomes for patients with infection/sepsis, including lactate [8,11,12,13], CRP [9], cluster of differentiation 64 [40], proadrenomedullin [41], interleukin-6 [9,10], procalcitonin [9,10,15,16,42], and many others [10,40,41,43]. The sensitivity of procalcitonin for diagnosing bacteremia overall (both Gram-positive and Gram-negative organisms) was reported to be 62% at a cutoff value of >0.5 µg/L, 76% at >0.25 µg/L, and 92% at >0.1 µg/L [21]. Therefore, the sensitivity of procalcitonin alone for bacteremia is not good enough for use as a rule-out test [21], and a procalcitonin measurement result should be interpreted in the context of the patient’s clinical information and data about the current medical condition, rather than being used as the single criterion for diagnosing a bacterial systemic infection [25].

Limitations also exist in the use of procalcitonin as a tool for the diagnosis of infection in oncologic emergencies. For example, there is still no consensus on the cutoff points to be used when interpreting procalcitonin levels, and established protocols differ by clinical setting (e.g., ICU vs. ED) [44]. Appropriate cutoff points for procalcitonin to diagnose bacterial systemic infection may also vary among different patient populations, and using two cutoff points (high and low cutoff points optimized for specificity or sensitivity) may be more clinically useful than using a single cutoff point for the diagnosis of systemic infection [45]. In the context of the current study, the biomarkers were used to determine prognosis and not for diagnosis of infection, and we found that using a single cutoff point provided the simplest regression model without compromising the performance of our final prediction model for determining prognosis.

Using this rationale, we were able to formulate a prediction score that can accurately guide patient treatment based on mortality risk. With an AUROC of 0.88, the prediction score appears to have excellent discrimination compared with other scoring systems, such as the Acute Physiology and Chronic Health Evaluation II score (0.714–0.828), Acute Physiology and Chronic Health Evaluation IV score (0.665–0.82), and Simplified Acute Physiology Score II (0.71–0.778) [46,47,48,49]. In addition, the absence of a poor fit in the Hosmer–Lemeshow statistic (0.837) indicates that the final prediction model was well-calibrated; no major discrepancy was noted between the observed and expected mortality rate predicted by the final model. The prediction score ranges from 0 to 21 and permits the stratification of patients with a cancer diagnosis into four categories correlating to the expected mortality rate. An important advantage of our final prediction model is that it includes basic clinical data recorded for all patients (qSOFA), as well as markers that are often routinely measured in patients with a cancer diagnosis (e.g., albumin, lactate dehydrogenase). The type of malignancy is also often overlooked in risk prediction scores, despite being readily available. The absence of these elements in traditional scores may put oncology patients at a disadvantage. This was particularly apparent in our cohort: 187 of the deaths that occurred in our study (79.0%) involved individuals with a qSOFA score ≤1. This reflects the inadequacy of traditional assessment systems in our target population. As such, adding clinical variables made our prediction score better tailored to patients with a cancer diagnosis. In addition, the multi-ethnic character of our population added to the external validity of the prediction score.

The ability to stratify patients by mortality risk holds great value for treating physicians, patients, and their families. In addition to assisting in triage and guiding patient disposition (ICU or regular ward), the prediction score gives physicians an accurate assessment to better optimize and personalize the patient’s management plan. This is particularly important because the cause of death in patients undergoing chemotherapy is often unrelated to the cancer itself, but rather to separate entities, such as infections, thromboembolic events, or metabolic derangements [50]. The prediction score may also be used by physicians and administrators for a more accurate performance assessment and benchmarking in a cancer treatment setting. Naturally, an accurate impression of a patient’s expected prognosis is important for patients and their families. Being aware of accurate statistics tailored to the patient’s population is vital in ensuring proper communication by physicians. Subsequently, this would lead to the creation of accurate expectations and goal setting by all involved parties.

Our study has some limitations. First, the prediction score was developed using values obtained only upon admission. Therefore, the final prediction model was not tested for sequential scoring of patients, and it remains to be seen whether a correlation exists between daily sequential assessment of patients with the prediction score and overall mortality. In addition, although several variables were considered during the formation of the prediction score, some factors could have been overlooked, such as the stage of the malignancy in question, rather than just the type. Given the retrospective nature of the study, information bias is possible, whereby some patients were not tested for all studied variables. As noted in the methods section, this risk was mitigated by excluding all variables that were not examined in at least 80% of patients. A risk of selection bias also exists given that the database used was from a single center. In addition, patients were considered to have bacteremia if they had any positive blood culture that was collected for suspected infection during the EC visit, regardless of the pathogen identified. Finally, it is important to note that despite the relatively large sample size used, some subcategories may have been underrepresented. For instance, only 14 patients presented with a qSOFA score of 3.

## 5. Conclusions

In summary, our study showed that lactate, CRP, and procalcitonin were independent predictors of short-term survival and ICU admission in cancer patients presenting to the ED with suspected infection/sepsis. We developed a new prediction score for clinical outcome that can be used in oncologic emergency settings for cancer patients with suspected infection/sepsis. This simple prediction score can stratify the risk of 14-day mortality, 30-day mortality, and ICU admission for each patient. A further prospective study is needed to validate this prediction score and assess its generalizability. The prediction score is a potentially important prognostic tool for emergency physicians to evaluate cancer patients with suspected infection/sepsis.

## Figures and Tables

**Table 1:** Demographic and clinical characteristics of cancer patients who visited the emergency department (ED) with suspected infection during the period studied.

Characteristic	No. of Patients (%)	No. of ED Visits (%)
Total	3623	5118
Age (interquartile range), years	62 (50–70)	62 (49–70)
Sex		
Female	1764 (48.7)	2433 (47.5)
Male	1859 (51.3)	2685 (52.5)
Race		
Nonwhite	1107 (30.6)	1636 (32.0)
White	2516 (69.4)	3482 (68.0)
Cancer type		
Leukemia	602 (16.6)	992 (19.4)
Lymphoma	394 (10.9)	597 (11.7)
Breast	330 (9.1)	432 (8.4)
Lung	313 (8.6)	390 (7.6)
Sarcoma	210 (5.8)	333 (6.5)
Multiple myeloma	202 (5.6)	322 (6.3)
Head and neck	181 (5.0)	257 (5.0)
Colorectal	163 (4.5)	202 (3.9)
Hepatobiliary	149 (4.1)	209 (4.1)
Male genital	142 (3.9)	186 (3.6)
Pancreatic	130 (3.6)	169 (3.3)
Uterine	108 (3.0)	138 (2.7)
Melanoma	91 (2.5)	106 (2.1)
Gastroesophageal	82 (2.3)	106 (2.1)
Ovarian	83 (2.3)	100 (2.0)
Kidney	69 (1.9)	82 (1.6)
Bladder	65 (1.8)	95 (1.9)
Thyroid	50 (1.4)	69 (1.3)
Brain and spinal cord	48 (1.3)	57 (1.1)
Others	211 (5.8)	276 (5.4)
Cancer stage		
Local/undetermined	755 (20.8)	1000 (19.5)
Hematologic	1198 (33.1)	1911 (37.3)
Advanced/metastatic	1670 (46.1)	2207 (43.1)

**Table 2:** Final prediction model used to derive the prediction score for 14-day mortality among cancer patients presenting to the emergency department with suspected infection.

Variable ^a^	Beta Coefficient	Standard Error	Odds Ratio (95% Confidence Interval)	*p*	Assigned Point(s)
qSOFA score					
0		Reference			
1	1.003	0.249	2.73 (1.67–4.45)	<0.001	3
2	1.382	0.349	3.98 (1.97–7.77)	<0.001	4
3	2.222	0.800	9.23 (1.87–46.03)	0.005	6
Procalcitonin ≥ 0.15 ng/mL	0.853	0.399	2.35 (1.13–5.52)	0.033	2
Lactate ≥ 2.0 mmol/L	1.153	0.234	3.17 (2.01–5.04)	<0.001	3
CRP ≥ 115 mg/L	0.535	0.241	1.71 (1.07–2.76)	0.026	1
LDH ≥ 285 U/L	1.295	0.245	3.65 (2.28–5.99)	<0.001	4
Albumin < 3.5 g/dL	1.124	0.287	3.08 (1.79–5.54)	<0.001	3
Cancer type risk group					
Low		Reference			
Intermediate	0.367	0.249	1.44 (0.88–2.34)	0.140	1
High	0.588	0.352	1.80 (0.87–3.51)	0.095	2

^a^ Abbreviations: qSOFA, quick sequential organ failure assessment; CRP, C-reactive protein; LDH, lactate dehydrogenase.

## Data Availability

The data presented in this study are available on request from the corresponding author. The data are not publicly available due and an IRB approval from MD Anderson Cancer Center is required to share the data.

## Supplementary Materials

The following are available online at https://www.mdpi.com/article/10.3390/cancers13164087/s1, Figure S1: Flow diagram for identifying study participants and determining study eligibility, Figure S2: Steps involved in the development of the final prediction score, Figure S3: Variation of procalcitonin levels by cancer stage, Figure S4: Variation of different infection biomarkers by blood culture gram stain, Figure S5: Area under the receiver operating characteristic curve (AUROC) analysis of the primary prediction model and the final prediction model, Figure S6: K-fold cross-validation accuracy of the final prediction model (K = 10), Figure S7. Association of final risk group with 3-month survival. Table S1: Fourteen-day mortality by quick sequential organ failure assessment (qSOFA) score for emergency department (ED) visits (*n* = 5118), Table S2: Variation of infection biomarkers by quick sequential organ failure assessment (qSOFA) score, Table S3: Variation of infection biomarkers by blood culture result, Table S4: Cancer type risk stratification, Table S5: Fourteen-day mortality rates stratified by prediction score, Table S6: Fourteen-day mortality rates stratified by prediction score risk group, Table S7: Thirty-day mortality and intensive care unit (ICU) admission rates stratified by prediction score risk group, Table S8: Univariate and multivariable Cox proportional hazards model analysis of three months survival for different final risk groups.

## References

[1] Hensley M.K., Donnelly J.P., Carlton E.F., Prescott H.C. (2019). Epidemiology and Outcomes of Cancer-Related versus Non-Cancer-Related Sepsis Hospitalizations. Crit. Care Med..

[2] Moore J.X., Akinyemiju T., Bartolucci A., Wang H.E., Waterbor J., Griffin R. (2020). Mediating Effects of Frailty Indicators on the Risk of Sepsis after Cancer. J. Intensive Care Med..

[3] Williams M.D., Braun L.A., Cooper L.M., Johnston J., Weiss R.V., Qualy R.L., Linde-Zwirble W. (2004). Hospitalized cancer patients with severe sepsis: Analysis of incidence, mortality, and associated costs of care. Crit. Care.

[4] Van de Louw A., Cohrs A., Leslie D. (2020). Incidence of sepsis and associated mortality within the first year after cancer diagnosis in middle aged adults: A US population based study. PLoS ONE.

[5] Ambrus J.L., Ambrus C.M., Mink I.B., Pickren J.W. (1975). Causes of death in cancer patients. J. Med..

[6] Inagaki J., Rodriguez V., Bodey G.P. (1974). Proceedings: Causes of death in cancer patients. Cancer.

[7] Nosari A., Barberis M., Landonio G., Magnani P., Majno M., Oreste P., Sozzi P. (1991). Infections in haematologic neoplasms: Autopsy findings. Haematologica.

[8] Freund Y., Delerme S., Goulet H., Bernard M., Riou B., Hausfater P. (2012). Serum lactate and procalcitonin measurements in emergency room for the diagnosis and risk-stratification of patients with suspected infection. Biomarkers.

[9] Fraunberger P., Wang Y., Holler E., Parhofer K.G., Nagel D., Walli A.K., Seidel D. (2006). Prognostic value of interleukin 6, procalcitonin, and C-reactive protein levels in intensive care unit patients during first increase of fever. Shock.

[10] Harbarth S., Holeckova K., Froidevaux C., Pittet D., Ricou B., Grau G.E., Vadas L., Pugin J., Geneva Sepsis N. (2001). Diagnostic value of procalcitonin, interleukin-6, and interleukin-8 in critically ill patients admitted with suspected sepsis. Am. J. Respir Crit. Care Med..

[11] Shapiro N.I., Howell M.D., Talmor D., Nathanson L.A., Lisbon A., Wolfe R.E., Weiss J.W. (2005). Serum lactate as a predictor of mortality in emergency department patients with infection. Ann. Emerg. Med..

[12] Wacharasint P., Nakada T.A., Boyd J.H., Russell J.A., Walley K.R. (2012). Normal-range blood lactate concentration in septic shock is prognostic and predictive. Shock.

[13] Jansen T.C., van Bommel J., Schoonderbeek F.J., Sleeswijk Visser S.J., van der Klooster J.M., Lima A.P., Willemsen S.P., Bakker J., group L.s. (2010). Early lactate-guided therapy in intensive care unit patients: A multicenter, open-label, randomized controlled trial. Am. J. Respir Crit. Care Med..

[14] Qu R., Hu L., Ling Y., Hou Y., Fang H., Zhang H., Liang S., He Z., Fang M., Li J. (2020). C-reactive protein concentration as a risk predictor of mortality in intensive care unit: A multicenter, prospective, observational study. BMC Anesth..

[15] Sbrana A., Torchio M., Comolli G., Antonuzzo A., Danova M., Italian Network for Supportive Care in O. (2016). Use of procalcitonin in clinical oncology: A literature review. New Microbiol..

[16] Gunasekaran V., Radhakrishnan N., Dinand V., Sachdeva A. (2016). Serum Procalcitonin for Predicting Significant Infections and Mortality in Pediatric Oncology. Indian Pediatr..

[17] Jin M., Khan A.I. (2010). Procalcitonin: Uses in the Clinical Laboratory for the Diagnosis of Sepsis. Lab. Med..

[18] Durnas B., Watek M., Wollny T., Niemirowicz K., Marzec M., Bucki R., Gozdz S. (2016). Utility of blood procalcitonin concentration in the management of cancer patients with infections. Onco. Targets Ther..

[19] Schuttrumpf S., Binder L., Hagemann T., Berkovic D., Trumper L., Binder C. (2006). Utility of procalcitonin concentration in the evaluation of patients with malignant diseases and elevated C-reactive protein plasma concentrations. Clin. Infect. Dis..

[20] Koizumi Y., Sakanashi D., Ohno T., Nakamura A., Yamada A., Shibata Y., Shiota A., Kato H., Hagihara M., Asai N. (2020). Plasma procalcitonin levels remain low at the onset of gram-positive bacteremia regardless of severity or the presence of shock: A retrospective analysis of patients with detailed clinical characteristics. J. Microbiol. Immunol. Infect..

[21] Goodlet K.J., Cameron E.A., Nailor M.D. (2020). Low Sensitivity of Procalcitonin for Bacteremia at an Academic Medical Center: A Cautionary Tale for Antimicrobial Stewardship. Open Forum. Infect. Dis..

[22] Karagiannis A.K., Girio-Fragkoulakis C., Nakouti T. (2016). Procalcitonin: A New Biomarker for Medullary Thyroid Cancer? A Systematic Review. Anticancer Res..

[23] Kataja A., Tarvasmaki T., Lassus J., Sionis A., Mebazaa A., Pulkki K., Banaszewski M., Carubelli V., Hongisto M., Jankowska E. (2021). Kinetics of procalcitonin, C-reactive protein and interleukin-6 in cardiogenic shock—Insights from the CardShock study. Int. J. Cardiol..

[24] Schneider R., Cohen M.J., Benenson S., Duchin O., Haviv Y.S., Elhalel-Darnitski M., Levin P.D. (2020). Procalcitonin in hemodialysis patients presenting with fever or chills to the emergency department. Intern. Emerg. Med..

[25] Lai L., Lai Y., Wang H., Peng L., Zhou N., Tian Y., Jiang Y., Gong G. (2020). Diagnostic Accuracy of Procalcitonin Compared to C-Reactive Protein and Interleukin 6 in Recognizing Gram-Negative Bloodstream Infection: A Meta-Analytic Study. Dis. Markers.

[26] Gu J.X., Zhang N., Li S.S., Zhang A.M., Yin Y., Li Y.F., Jia M. (2020). The detection of bacterial infections in leukemia patients using procalcitionin levels. Leuk Lymphoma.

[27] Ito A., Ito I., Inoue D., Marumo S., Ueda T., Nakagawa H., Taki M., Nakagawa A., Tatsumi S., Nishimura T. (2020). The utility of serial procalcitonin measurements in addition to pneumonia severity scores in hospitalised community-acquired pneumonia: A multicentre, prospective study. Int. J. Infect. Dis..

[28] Arulkumaran N., Khpal M., Tam K., Baheerathan A., Corredor C., Singer M. (2020). Effect of Antibiotic Discontinuation Strategies on Mortality and Infectious Complications in Critically Ill Septic Patients: A Meta-Analysis and Trial Sequential Analysis. Crit. Care Med..

[29] DeSear K.E., Thompson-Leduc P., Kirson N., Chritton J.J., Ie S., Van Schooneveld T.C., Cheung H.C., Ou S., Schuetz P. (2020). ProCommunity: Procalcitonin use in real-world US community hospital settings. Curr. Med. Res. Opin..

[30] Langford B.J., Beriault D., Schwartz K.L., Seah J., Pasic M.D., Cirone R., Chan A., Downing M. (2020). A real-world assessment of procalcitonin combined with antimicrobial stewardship in a community ICU. J. Crit. Care.

[31] Lafon T., Cazalis M.A., Vallejo C., Tazarourte K., Blein S., Pachot A., Laterre P.F., Laribi S., Francois B., The TRIAGE Study Group (2020). Prognostic performance of endothelial biomarkers to early predict clinical deterioration of patients with suspected bacterial infection and sepsis admitted to the emergency department. Ann. Intensive Care.

[32] Haag E., Molitor A., Gregoriano C., Muller B., Schuetz P. (2020). The value of biomarker-guided antibiotic therapy. Expert Rev. Mol. Diagn..

[33] Li W., Wang M., Zhu B., Zhu Y., Xi X. (2020). Prediction of median survival time in sepsis patients by the SOFA score combined with different predictors. Burn. Trauma.

[34] Xia Y., Zou L., Li D., Qin Q., Hu H., Zhou Y., Cao Y. (2020). The ability of an improved qSOFA score to predict acute sepsis severity and prognosis among adult patients. Medicine.

[35] LeGuen M., Ballueer Y., McKay R., Eastwood G., Bellomo R., Jones D., Austin Health R.R.T.q.i. (2018). Frequency and significance of qSOFA criteria during adult rapid response team reviews: A prospective cohort study. Resuscitation.

[36] Singer M., Deutschman C.S., Seymour C.W., Shankar-Hari M., Annane D., Bauer M., Bellomo R., Bernard G.R., Chiche J.D., Coopersmith C.M. (2016). The Third International Consensus Definitions for Sepsis and Septic Shock (Sepsis-3). JAMA.

[37] Vincent J.L., de Mendonca A., Cantraine F., Moreno R., Takala J., Suter P.M., Sprung C.L., Colardyn F., Blecher S. (1998). Use of the SOFA score to assess the incidence of organ dysfunction/failure in intensive care units: Results of a multicenter, prospective study. Working group on “sepsis-related problems” of the European Society of Intensive Care Medicine. Crit. Care Med..

[38] Seymour C.W., Liu V.X., Iwashyna T.J., Brunkhorst F.M., Rea T.D., Scherag A., Rubenfeld G., Kahn J.M., Shankar-Hari M., Singer M. (2016). Assessment of Clinical Criteria for Sepsis: For the Third International Consensus Definitions for Sepsis and Septic Shock (Sepsis-3). JAMA.

[39] Zweig M.H., Campbell G. (1993). Receiver-operating characteristic (ROC) plots: A fundamental evaluation tool in clinical medicine. Clin. Chem..

[40] Gibot S., Bene M.C., Noel R., Massin F., Guy J., Cravoisy A., Barraud D., De Carvalho Bittencourt M., Quenot J.P., Bollaert P.E. (2012). Combination biomarkers to diagnose sepsis in the critically ill patient. Am. J. Respir Crit. Care Med..

[41] Henriquez-Camacho C., Losa J. (2014). Biomarkers for sepsis. Biomed. Res. Int..

[42] Garcia de Guadiana-Romualdo L., Cerezuela-Fuentes P., Espanol-Morales I., Esteban-Torrella P., Jimenez-Santos E., Hernando-Holgado A., Albaladejo-Oton M.D. (2019). Prognostic value of procalcitonin and lipopolysaccharide binding protein in cancer patients with chemotherapy-associated febrile neutropenia presenting to an emergency department. Biochem. Med..

[43] Yang Z., Qdaisat A., Hu Z., Wagar E.A., Reyes-Gibby C., Meng Q.H., Yeung S.C. (2016). Cardiac Troponin Is a Predictor of Septic Shock Mortality in Cancer Patients in an Emergency Department: A Retrospective Cohort Study. PLoS ONE.

[44] Schuetz P., Beishuizen A., Broyles M., Ferrer R., Gavazzi G., Gluck E.H., Gonzalez Del Castillo J., Jensen J.U., Kanizsai P.L., Kwa A.L.H. (2019). Procalcitonin (PCT)-guided antibiotic stewardship: An international experts consensus on optimized clinical use. Clin. Chem. Lab. Med..

[45] Peacock W.F., Rafique Z. (2020). Procalcitonin Cut-point Strategies. Clin. Infect. Dis..

[46] Czajka S., Ziebinska K., Marczenko K., Posmyk B., Szczepanska A.J., Krzych L.J. (2020). Validation of APACHE II, APACHE III and SAPS II scores in in-hospital and one year mortality prediction in a mixed intensive care unit in Poland: A cohort study. BMC Anesth..

[47] Gilani M.T., Razavi M., Azad A.M. (2014). A comparison of Simplified Acute Physiology Score II, Acute Physiology and Chronic Health Evaluation II and Acute Physiology and Chronic Health Evaluation III scoring system in predicting mortality and length of stay at surgical intensive care unit. Niger Med. J..

[48] Varghese Y.E., Kalaiselvan M.S., Renuka M.K., Arunkumar A.S. (2017). Comparison of acute physiology and chronic health evaluation II (APACHE II) and acute physiology and chronic health evaluation IV (APACHE IV) severity of illness scoring systems, in a multidisciplinary ICU. J. Anaesthesiol Clin. Pharm..

[49] Almansour I.M., Aldalaykeh M.K., Saleh Z.T., Yousef K.M., Alnaeem M.M. (2020). Predictive Performance of two Measures of Prognostic Mortality of Cancer Patients in Intensive Care Unit in Jordan: A Comparative Single-Centre Study. Open Nurs. J..

[50] O’Brien M.E., Borthwick A., Rigg A., Leary A., Assersohn L., Last K., Tan S., Milan S., Tait D., Smith I.E. (2006). Mortality within 30 days of chemotherapy: A clinical governance benchmarking issue for oncology patients. Br. J. Cancer.

