# Supplementary material for: Prognostic Value of Procalcitonin, C-Reactive Protein, and Lactate Levels in Emergency Evaluation of Cancer Patients with Suspected Infection
Source: Cancers (Basel). 2021 Aug 13;13(16):4087. doi: 10.3390/cancers13164087 (PMC8393196; doi:10.3390/cancers13164087)
Supplement: Supplementary file 1 [file cancers-13-04087-s001.zip › cancers-1281651-SI.pdf]

cancers

MDPI

Article

Prognostic Value of Procalcitonin, C-Reactive Protein, and Lactate Levels in Emergency Evaluation of Cancer Patients with Suspected Infection

Patrick Chaftari, Aiham Qdaisat, Anne-Marie Chaftari, Julian Maamari, Ziyi Li, Florea Lupu, Issam Raad, Ray Hachem, George Calin and Sai-Ching Jim Yeung

Figures

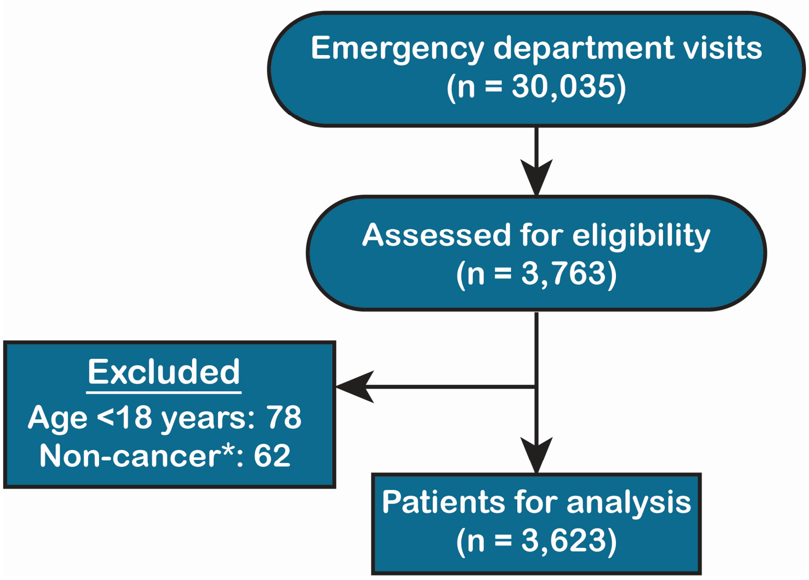

Figure S1. Flow diagram for identifying study participants and determining study eligibility. *"Non-cancer" indicates patients who did not have a confirmed cancer diagnosis prior to the emergency department visit.

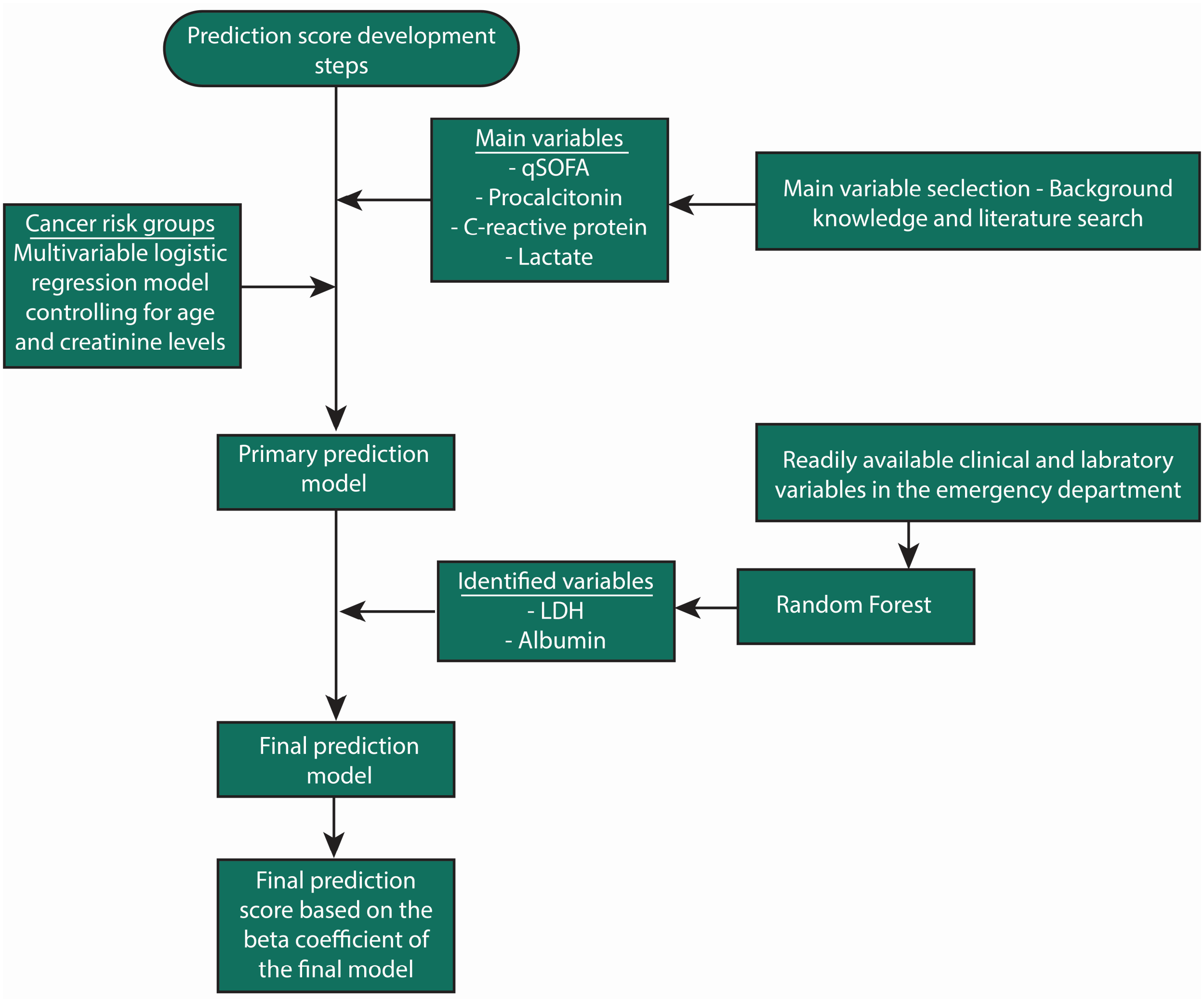

Figure S2. Steps involved in the development of the final prediction score. qSOFA, quick sequential organ failure assessment; LDH, lactate dehydrogenase.

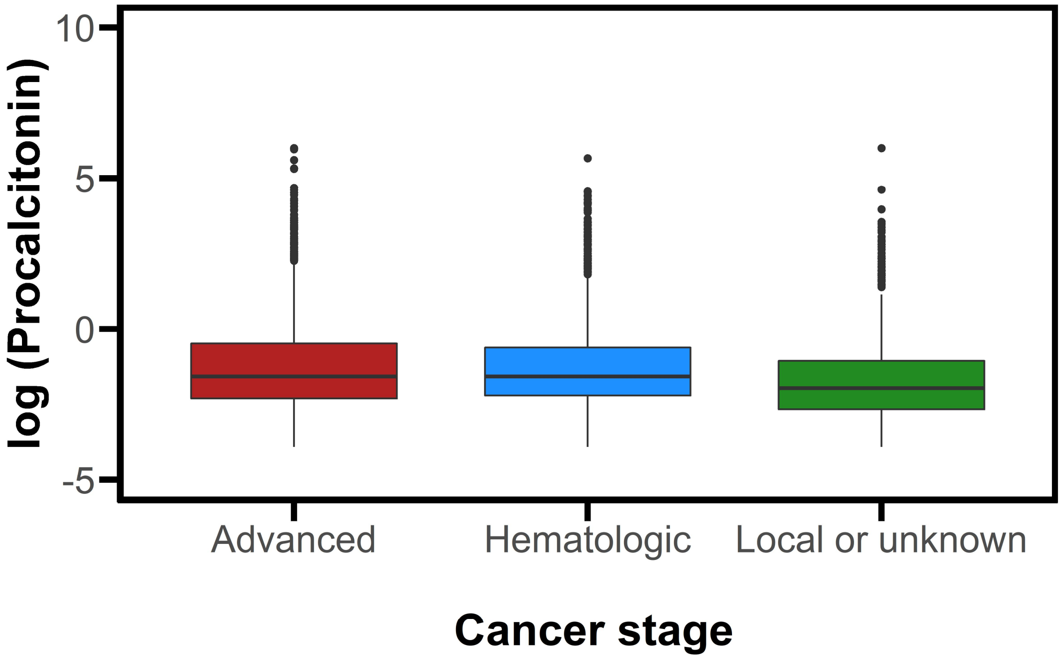

Figure S3. Variation of procalcitonin levels by cancer stage.

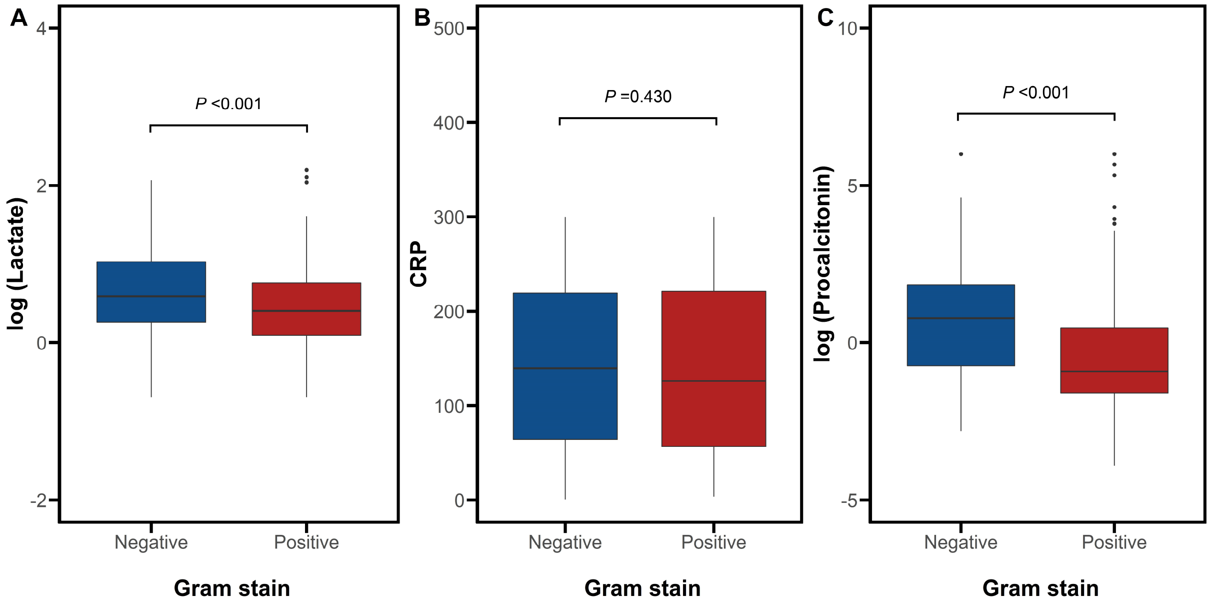

Figure S4. Variation of different infection biomarkers by blood culture gram stain.

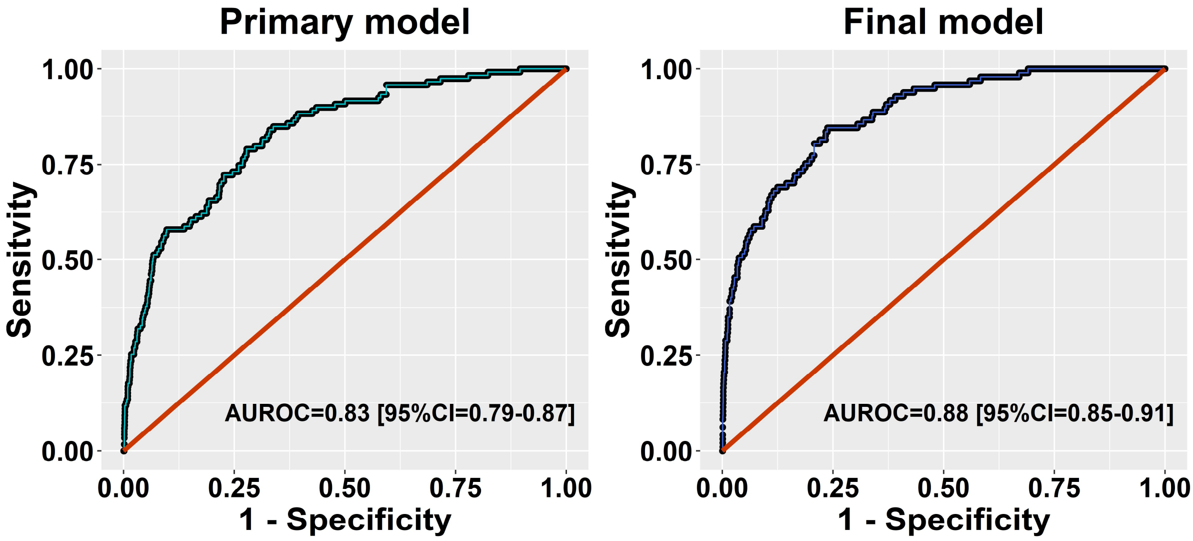

Figure S5. Area under the receiver operating characteristic curve (AUROC) analysis of the primary prediction model and the final prediction model. Abbreviations: CI, confidence interval.

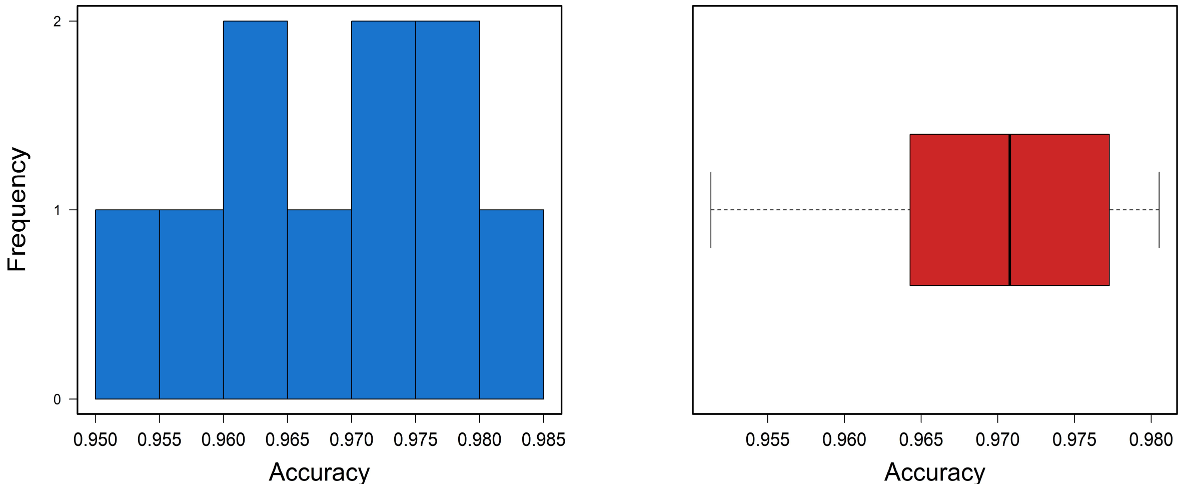

Figure S6. K-fold cross-validation accuracy of the final prediction model (K = 10).

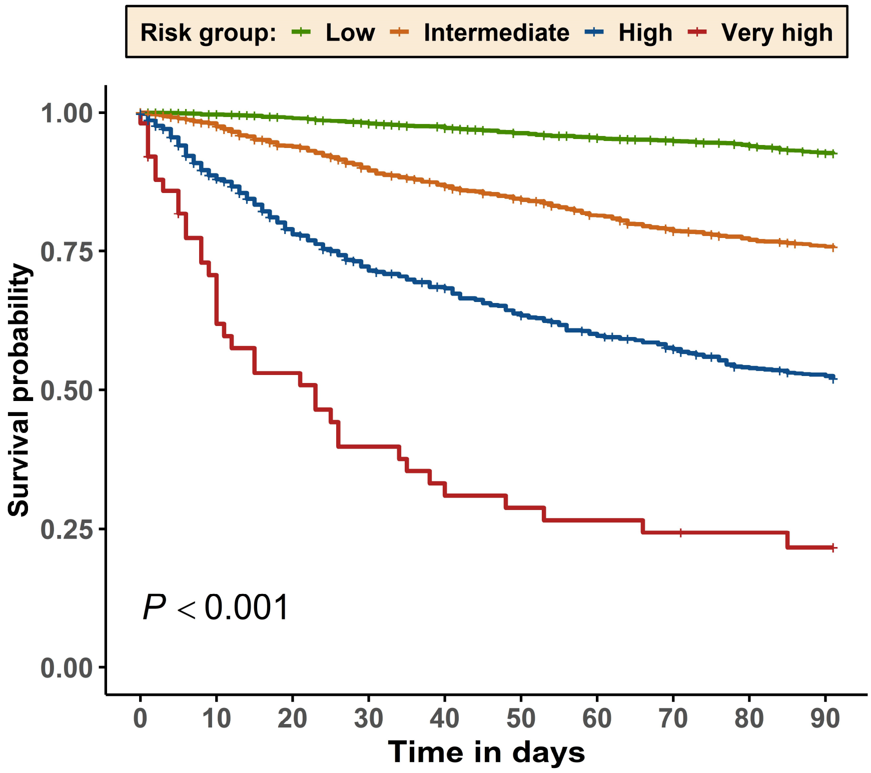

Figure S7. Association of final risk group with 3-month survival. Kaplan-Meier curves for 3-month (90-day) survival are shown for each risk groups (low risk, intermediate risk, high risk, and very high risk).

Tables

Table S1. Fourteen-day mortality by quick sequential organ failure assessment (qSOFA) score for emergency department (ED) visits (n = 5118).

qSOFA Score	No. of ED Visits (%)	14-Day Mortality	
		No	Yes
0	3834 (74.9)	3757	77
1	1078 (21.1)	968	110
2	192 (3.8)	162	30
3	14 (0.3)	7	7

Table S2. Variation of infection biomarkers by quick sequential organ failure assessment (qSOFA) score.

Biomarker[a]	qSOFA Score				P
	0	1	2	3	
Median (IQR) lactate, mmol/L	1.30 (1.00-1.80)	1.60 (1.10-2.30)	2.10 (1.40-2.98)	4.20 (3.40-4.90)	<0.001
Median (IQR) CRP, mg/L	67.1 (19.3-137.6)	94.2 (39.1-180.7)	120.5 (50.8-227.4)	257.6 (178.4-300.0)	<0.001
Median (IQR) procalcitonin, ng/mL	0.16 (0.09-0.38)	0.32 (0.13-0.99)	0.91 (0.25-5.39)	2.70 (1.55-41.86)	<0.001

[a]Abbreviations: IQR, interquartile range; CRP, C-reactive protein.

Table S3. Variation of infection biomarkers by blood culture result.

Biomarker[a]	Blood Culture		P
	Negative	Positive	
Median (IQR) lactate, mmol/L	1.40 (1.00-1.90)	1.70 (1.10-2.50)	<0.001
Median (IQR) CRP, mg/L	67.3 (19.6-140.1)	130.2 (59.3-220.9)	<0.001
Median (IQR) procalcitonin, ng/mL	0.17 (0.09-0.43)	0.89 (0.28-3.99)	<0.001

[a]Abbreviations: IQR, interquartile range; CRP, C-reactive protein.

Table S4. Cancer type risk stratification.[a]

Variable Name	AOR[b]	95% CI	Final Risk Group
Gastroesophageal	2.18	0.85-5.21	High risk
Hepatobiliary and pancreatic	2.05	1.08-4.01	High risk
Lung	1.97	1.04-3.88	Intermediate risk
Gynecologic	1.31	0.59-2.87	Intermediate risk
Melanoma	1.40	0.49-3.56	Intermediate risk
Urinary	1.25	0.51-2.88	Intermediate risk
Breast	1.23	0.62-2.51	Intermediate risk
Colorectal	1.20	0.51-2.73	Intermediate risk
Male genital	0.98	0.38-2.33	Low risk
Leukemia	0.92	0.49-1.79	Low risk
Head and neck	0.83	0.33-1.94	Low risk
Lymphoma	0.60	0.28-1.28	Low risk
Multiple myeloma	0.49	0.18-1.19	Low risk
Sarcoma	0.43	0.14-1.13	Low risk

[a]Abbreviations: AOR, adjusted odds ratio; CI, confidence interval. [b]AOR adjusted for age and creatinine level.

Table S5. Fourteen-day mortality rates stratified by prediction score.

Prediction Score	Total	14-Day Mortality		Mortality Rate, %
		No	Yes	
0	568	566	2	0.4
1	360	358	2	0.6
2	396	395	1	0.3
3	499	497	2	0.4
4	425	422	3	0.7
5	421	419	2	0.5
6	514	499	15	2.9
7	345	334	11	3.2
8	262	247	15	5.7
9	366	345	21	5.7
10	287	257	30	10.5
11	144	130	14	9.7
12	167	144	23	13.8
13	130	106	24	18.5
14	80	65	15	18.8
15	53	44	9	17
16	50	36	14	28
17	29	20	9	31
18	11	6	5	45.5
≥19	11	4	7	63.6

Table S6. Fourteen-day mortality rates stratified by prediction score risk group.

Risk Group	Cutoff Point for Prediction Score	14-day Mortality		Mortality Rate, %
		No	Yes	
Low risk	≤5	2657	12	0.4
Intermediate risk	6-9	1425	62	4.2
High risk	10-15	782	129	14.2
Very high risk	≥16	30	21	41.2

Table S7. Thirty-day mortality and intensive care unit (ICU) admission rates stratified by prediction score risk group.

Final Risk Group	30-Day Mortality	ICU Admission
Low risk (≤5 points)	46/2669 (1.7%)	10/2669 (0.4%)
Intermediate risk (6-9)	143/1487 (9.6%)	60/1487 (4.0%)
High risk (10-15 points)	233/911 (25.6%)	117/911 (12.8%)
Very high risk (≥16 points)	29/51 (56.9%)	21/51 (41.2%)

Table S8. Univariate and multivariable Cox proportional hazards model analysis of 3-month survival for each final risk group.

Variable	Univariate		Multivariable	
	HR (95% CI)	*P*	HR* (95% CI)	*P*
Final score risk group				
Low risk	Reference		Reference	
Intermediate risk	3.64 (3.05-4.35)	<0.001	3.59 (3.00-4.28)	<0.001
High risk	8.94 (7.53-10.62)	<0.001	8.81 (7.42-10.48)	<0.001
Very high risk	25.06 (17.53-35.83)	<0.001	24.90 (17.38-35.67)	<0.001

HR, hazard ratio; CI, confidence interval. * Controlling for age, sex, and race.
